# 

**Published:** 1914-07-04

**Authors:** 


					Southampton Street Strand, London W.O.

				

